# Behind the good of digital tools for occupational safety and health: a scoping review of ethical issues surrounding the use of the internet of things

**DOI:** 10.3389/fpubh.2024.1468646

**Published:** 2024-12-20

**Authors:** Maéva El Bouchikhi, Sophie Weerts, Christine Clavien

**Affiliations:** ^1^Swiss Graduate School of Public Administration, University of Lausanne, Lausanne, Switzerland; ^2^Institute For Ethics, History, and the Humanities, Faculty of Medicine, University of Geneva, Geneva, Switzerland

**Keywords:** internet of things, ethical issues, occupational safety and health, opportunities, surveillance, adverse effects

## Abstract

**Introduction:**

The internet of things (IoT) is increasingly used for occupational safety and health (OSH) purposes in private and public organisations. Current practices and regulations are unclear, and some stakeholders raised concerns about deploying this technology at work.

**Methods:**

Following the PRISMA-ScR checklist, we reviewed the main opportunities and ethical issues raised by using IoT devices for OSH purposes, as discussed in the academic literature. We searched peer-reviewed papers from 2008 to September 2023, written in English and available in “Web of Science,” “PhilPapers,” and “Google Scholar.” We found 1,495 articles, of which 61 fulfilled the selection criteria. We classified ethical topics discussed in the papers in a coherent description frame.

**Results:**

We obtained 6 overarching categories: “ethical opportunities,” “surveillance and problematic data re-purposing,” “difficulty to inform, consult, and obtain consent from employees,” “unintended and unpredictable adverse effects,” “suboptimal data management,” and “external factors that are conducive to ethical issues.” The resulting list of problematic issues is unexpectedly furnished and substantial. Such a list provides information and guidance for those who wish to develop evaluation frameworks in line with a preventive regulatory approach. It also informs policymakers and practitioners about the governance of such tools for ensuring more OSH.

## 1 Introduction

Private and public organisations increasingly deploy connected devices such as sensors, that continuously collect and communicate data. These devices, also called Internet of things (IoT), often involve algorithmic systems and complex AI technology. They can contribute to automating arduous tasks, optimise the use of space, monitor employees’ pace of work, or even reduce the risks of work-related accidents and diseases. They may help to detect early burnout, support healthy lifestyles, promote well-being, facilitate medical treatment or detect disease transmission in an epidemic context (1). They may also take a great variety of forms, such as connected chairs, smart watches for corporate wellness programmes, stress sensors, risky behaviour trackers (e.g., fast driving), safety material (e.g., helmets, boots) with built in GPS, or contact tracing devices (2, 3). Their contribution to better health and more safety at work allows them to be characterised as tools for fostering the agenda for decent work promoted by international organisations.

However, media, international agencies, non-profit organisations and scholars warn of problems related to the deployment of technological solutions, including IoTs, in the workplace and beyond. For example, in 2016, the Guardian released that Uber experienced a massive data breach after having extensively monitored and collected the personal data of its employees (4). In 2023, a scandal broke out at Queen Mary University in London, because space utilisation tracking tools were deployed without consulting faculty members and students (5). The *European Agency for Safety and Health at Work* highlighted that the adverse effects of hidden surveillance count among the main future challenges posed by IoTs, automation of tasks, artificial intelligence, and autonomous decision-making systems (6–8). Similarly, the *European Digital Rights* (EDRi) and the non-profit-organisation *Worker Info Exchange* (WIE) report evidence that traditional and gig economy workers are more controlled, managed, and subject to algorithmic surveillance through sensors, facial recognition, and other automated-decision-making systems than before (9). All these elements highlight the reinforcement of a surveillance society, exacerbated in the workplace through the deployment of algorithmic management (10, 11).

Yet the risks of using IoT for occupational safety and health (OSH) have remained a blind spot in policy and academic work. At the level of regulation, lawmakers have been sensitive to numerous challenges posed by digital transformation at work while overlooking the risks of IoT deployed for OSH. For example, the European Union has categorised as “high-risk” some types of AI systems deployed for optimising employment, worker management or access to self-employment. Precautionary measures and transparency obligations have recently been enforced in the new regulation, imposing limits on the devices that can be put on the market or used to make decisions on recruitment, promotion, termination of contractual relationships or task allocation based on algorithmic analysis of individual behaviour or personal traits or characteristics (12). Similarly, in the literature, although the challenges posed by the digitalization of the workplace are largely debated, little research is specifically dedicated to the acceptability of IoT for OSH purposes. Relevant issues are addressed, but mostly in passing or not in a structured and targeted way.

We consider it as important to make a sound and full-fledged critical assessment of any information technology before deploying it. For such a task, it is important to foster fundamental knowledge about the ethical pros and cons of deploying IoTs for OSH purposes. For this, we conducted two studies: a scoping review, presented here, and a qualitative research including stakeholders, published separately (36).

This paper offers a comprehensive list of the benefits and ethical issues raised by IoTs for OSH. This fundamental knowledge should provide the necessary understanding for building robust and practical ethical frameworks. Moreover, it should enlighten policymakers regarding any generalisation of digital tools for implementing OSH policy and their evaluation methods. Finally, it should help stakeholders engaged in labour and OSH policies: employers, unions and OSH practitioners.

In the next section, we describe the methods we used to conduct our scoping review. We then present our results, which we classified into benefits and negative impacts. We follow up with a discussion section on the main common trends among this variety of problems. In conclusion, we draw future research avenues.

## 2 Methods

To conduct our scoping review, we followed the PRISMA-ScR checklist (13). We searched in “Web of Science,” “PhilPapers,” and “Google Scholar” for articles, proceedings, or editorials, published or accepted in early access, after 2008, the date of the important inaugural international *Internet of Things* conference (14, 15). We choose these three databases because together, they enable the identification of high quality and influential articles produced in the fields of science and technology, philosophy and ethics, and the social sciences.

Since there is no established or standardised term for our topic of investigation, we started with a broad search strategy, in order to identify relevant synonyms and spelling variations on relevant key terms covering the topics of ethics, IoTs and OSH. We notably used “ethic*,” “ethics,” “internet of things,” “IoT,” “technology,” “artificial intelligence,” “big data,” “AI,” “wearable,” “health monitoring,” “algorithms,” “workplace,” “work,” “occupational health,” “safety,” “health.” We used these keywords with OR and AND relations and adapted our search queries to the features and content of the databases (see Supplementary File 1 for details). Research and selection criteria were elaborated collaboratively between the authors. The first author conducted the search and selected the articles. The second and third authors double checked the selected articles.

We selected the articles based on the quality of the journal, and on the content of the title, abstract, and a quick key-word search in the main text in case of doubt. For this, we applied the following selection criteria: the article [is published in an English peer-reviewed journal (to ensure minimal academic quality)] *AND* [discusses ethical opportunities and issues of IoT] *AND* [includes an explicit discussion on OSH] *OR* [discusses with some detail at least one case of IoT used for OSH purposes]. Since our research topic is rarely discussed as such in the literature, we had to keep our selection criteria broad enough in order to find relevant articles. Moreover, while reading the selected article, we included additional articles fulfilling our selection criteria based on snowballing research around the most cited or relevant items that we found (16).

We read the selected articles and took note, in a common file, of the opportunities and ethical issues that can relevantly be applied to IoTs for OSH purposes. The first author read all the selected articles, while the second and third authors read a subsection of the material. 50% of the material was read by at least two authors. In a common file, the topics were progressively added by all authors while the third author grouped and described them in overarching categories, composed of subtopics. Categories and subtopics were reorganised until we obtained a logically coherent description frame. This work was done in an interactive process involving all authors. There is no pre-registered protocol for this scoping review.

## 3 Results

The database search was carried out from August to September 2023. 1,495 articles were found, of which 47 items were accessible and fulfilled our selection criteria (see Supplementary File 1 for details). Snowballing research added 14 further items to the list, bringing the total to 61 selected articles (see Table 1). We could not access 8 articles that seemed to fulfil our research criteria based on reading their abstract. Paywalls blocked these articles, and their authors did not respond to our request to send us a copy.

**Table 1:** List of selected articles, numbered as they are cited in the result section.

1.	Khakurel et al. (49)	2.	Chan et al. (33)	3.	Ajunwa et al. (20)
4.	Yassaee et al. (79)	5.	Naous and Mettler (2)	5.	Schall et al. (80)
7.	Maltseva (54)	8.	Richardson and Mackinnon (81)	9.	Moore (66)
10.	Lupton (52)	11.	Jacobs et al. (43)	12.	Ajana (19)
13.	Gabriels and Coeckelbergh (38)	14.	Moore (15)	15.	Moore and Piwek (67)
16.	Moore (68)	17.	Bovens et al. (26)	18.	Marinescu et al. (55)
19.	Burr et al. (29)	20.	Mettler and Stepanovic (59)	21.	Mettler and Stepanovic (60)
22.	Brous and Janssen (28)	23.	Baldassarre et al. (22)	24.	Pouyakian (73)
25.	Mejia et al. (62)	26.	Leclercq-Vandelannoitte (51)	27.	Tucker et al. (82)
28.	Costantino et al. (34)	29.	Calvard (30)	30.	Mcaleenan et al. (61)
31.	Molaei et al. (65)	32.	Oravec (83)	33.	Sestino et al. (84)
34.	Tamers et al. (85)	35.	Abioye et al. (18)	36.	Bavaresco et al. (24)
37.	Patel et al. (86)	38.	Svertoka et al. (87)	39.	Suder and Siibak (88)
40.	Pütz et al. (89)	41.	Weerts et al. (90)	42.	Cebulla et al. (31)
43.	Chalaris (32)	44.	Jetha et al. (45)	45.	Segkouli et al. (91)
46.	Sharon (92)	47.	Malomane et al. (53)	48.	Kabir and Alam (46)
49.	Bowen et al. (27)	50.	Pasquale et al. (93)	51.	Howard (41)
52.	Six Dijkstra (94)	53.	Le Feber et al. (50)	54.	Segura et al. (95)
55.	Iavicoli et al. (42)	56.	Martinetti et al. (56)	57.	Nihan (96)
58.	Niknejad et al. (97)	59.	Martinez-Martin et al. (58)	60.	Martinez-Martin et al. (57)
61.	Karale (47)				

During the analysis process, we approached saturation of new relevant topics discussed in the literature after reading 40–45 articles. Here we provide a summary of the main topics identified. We grouped them into 6 overarching categories composed of subtopics and described them with some illustrative references. The resulting description frame is summarised in Table 2.

**Table 2:** Overview of topics discussed in the literature.

Ethical opportunities	
Surveillance and problematic data-repurposing	Surveillance
	Re-use for dubious purposes
Lack of information, participation, and consent	Lack of participation
	Lack of informed and free consent
	Abuse of trust
Unintended and unpredictable adverse effects	Negative impacts on employees’ physical and mental health
	Weakening of workers’ power, competencies and self-confidence
	Negative impacts on working processes and habits
	Risks of breaches of sensitive information
	Pressure for normalisation and exacerbation of social stigma
	New or increased existing inequalities
	Weakening of human relationships at work
	Weakening of trust relationships at work
	De-responsibilisation of employers
	Normalisation of surveillance practices
	Dystopian and dehumanised workplaces
	Increased controversies surrounding IoTs and AI in public discourses
	Disruption drivers of public health and employment policies
	Negative environmental impact
Suboptimal data management	More data than needed are collected or stored in the long term
	A too large spectrum of stakeholders has access to the data
	Lack of secure systems
	Low quality of data or of data analysis
	Numerous devices deployed in the workplace
Contextual factors that are conducive to ethical issues	Blurred limits between professional and private life
	Techno-optimism and lack of ethical awareness among stakeholders
	Emergency situations
	Complexity and lack of competencies
	Lack of scientific evidence or peer-reviewed data
	Costs of ethical safeguards
	Lack of clear overall governance

### 3.1 Ethical opportunities

IoTs for OSH purposes can generate ethical opportunities for workers, employers, and society. First, these devices may positively contribute to preventing work-related safety risks and health problems. They may diffuse useful and timely information, inputs, and counselling to workers, leading them to become more aware of OSH risks, to gain knowledge on themselves (i.e., early illness detection, identification of weaknesses or bad habits), to increase their individual responsibility over health, to empower them to set and achieve personal health goals, to take more care of their safety, to gain assurance in their relationship with health professionals, and possibly with researchers working with their data. Some IoTs provide the possibility to reach out to others for helpful counselling or debriefing, to communicate and facilitate exchanges with those who share similar hardships, or to exchange experiences. These interactions enforce solidarity, socialisation, and community experience. IoTs may also reduce the burden of physical and unsafe tasks, train and nudge workers to adopt safe and healthy working habits, or help optimise their work environment, an asset for maintaining healthy workers. In specific contexts, besides health and safety, some IoTs may provide employees space for more autonomy or flexibility (e.g., working from home may otherwise not be allowed). From these points of view, IoTs can serve as effective means to decrease fatalities, to improve health prevention, workers’ capabilities and productivity at work, work-life balance, and global and well-being and healthy lifestyle^(1–49)^.

These devices are also useful for employers that will be more aware of non-optimal working conditions (e.g., information about stress levels at work or about factors that increase the probability of hazards). Indeed, through the data collection and analysis process, IoTs make it possible to make decisions or formulate recommendations that are more objective, accurate, equitable, and free of prejudices because they are based on a large amount of data collected continuously and analysed in a standardised way, a mark of reliability ^(7,9,23,35,43,44,46,52,53)^. Such information will help employers to enhance the quality of risk assessments and diagnosis of OSH situations, simplify the management of risk avoidance measures, optimise emergency responses, facilitate accountability in case of injuries, assess and demonstrate the efficiency of OSH measures ^(1,4,5,7,11,15,17,20-23,25,28,31,34,35,38,40,41,43-47,49-51)^. Moreover, IoTs can help employers identify vulnerable groups in specific conditions and tailor subsequent interventions for them ^(21,23,25,34,49)^.

Finally, the deployment of IoTs may have several large-scale societal benefits. If broadly deployed in multiple companies, they can help curb the dissemination of a contagious illness or alert to the outbreak of an epidemic risk. Aggregated information can also contribute to research, with positive downstream effects on public health, clinical practices, and the optimisation of healthcare delivery and OSH practices ^(10,19,23,34,46)^. These devices may also contribute to optimise workflows and reduce the costs related to medical care ^(12,43)^.

### 3.2 Surveillance and problematic data-repurposing

Surveillance and information re-purposing is the first set of ethical issues that the deployment of IoTs for OSH purposes can generate.

#### 3.2.1 Surveillance

Authors express strong worries about the hidden surveillance made possible with IoTs and autonomous decision-making systems ^(9,41)^. “*The way these technologies are used in organizations has led to the development of new forms of control, which seem more insidious, subtle, and misleading than past forms of IT-based control* (e.g.*, computerized performance monitoring*)*, in that they are less visible, are indirect, and are often disguised with a rhetoric of emancipation and autonomy*.” ^(26)^. IoTs allow employers to collect, visualise, and analyse large amounts of personal and sensitive worker data ^(5,10,21,31,48,54)^. This massive data collection enables performance analyses and the monitoring of workers and is conducive to surveillance practices that benefit companies, such as excessive managerial control or problematic intrusion in employees’ privacy ^(1-7,9,12-16,18-21,24-26,29-32,46,48,49,51,55-58)^. For instance, some taxi companies have implemented sensors to detect factors that endanger safe driving (e.g., prolonged working hours, speed, and signs of driver fatigue). These devices help avoid car accidents but can also be used to detect and fire poor drivers ^(42)^. In this context, IoTs increase the asymmetric power relationship between employers and workers, especially the most vulnerable ^(1,3,29,40,52)^.

Surveillance becomes ubiquitous with IoTs that can also be easily relocated and capable of collecting a wide range of data. Once one form of justifiable surveillance (e.g., a necessary means for sending individualised safety warnings related to a work task) is enforced and accepted, it can be abusively extended beyond its original scope (e.g., for controlling workers’ pace of work) ^(5,7,48)^. In extreme cases where data become enmeshed in larger networks, by promoting the use of IoTs for OSH purposes, private companies may contribute to some forms of State and international surveillance, as exemplified by contract tracing apps connected to datacentres of national agencies ^(10,16,29,39,46,48,54)^.

#### 3.2.2 Re-use for dubious purposes

Even in the case of IoTs primarily deployed for OSH purposes without surveillance goals, once the collected data are at disposal, they still may be *re-used for dubious purposes*. This risk of “function creep” (expansion of the use of a system or technology beyond its original purposes) is high considering the ambiguous interest of companies: they need workers in good health while expecting them to be loyal and productive. In this respect, IoTs provide extensive ways to collect information and monitor workers’ behaviour or performance, sometimes without their awareness ^(3,5,7,12,14-18,20,21,23,24,26,30,39-43,45,46,48,49,51,55)^. For instance, a manager disposing of a flux of data on individual employees’ locations, originally used for contact tracing to combat the COVID-19 pandemic, may be tempted to check an employee’s attendance at work or to verify whether one particular employee is telling the truth when claiming compensation for injuries while working. In another example, early indicators of chronic disease revealed by a wellness-promotion device are used for discriminatory firing, task ascription or promotion practices ^(1,3,7,12,18,20,39,40,53)^. Data can also be transmitted or bought by third-party companies, or State administration ^(3,10,12,18,25,27,30,32,39,41,42,52,54,55,59)^. Some authors also point out the issue of “free labour,” designating unpaid transmission of sensitive information to third parties that benefit from it ^(4,5,15,46)^.

However, the dual use or re-purposing of data is not necessarily considered problematic, notably when used for valuable goals such as research or quality control. In such a case, these secondary goals, and the means to achieve them (i.e., who has access to what) must be properly assessed and justified, notably by showing that such type of re-purposing is likely to be generally accepted, including by the first users of the devices, or by showing that the users can benefit from the products or services resulting from the secondary use of their data ^(30,59)^.

### 3.3 Lack of information, participation, and consent

A further group of topics discussed in the literature is related to respect for employees’ points of view. As primary beneficiaries of OSH measures and directly concerned by the surveillance dimension of IoTs, their opinion and consent are of first importance. However, the scholarship shows that such a point is often not undertaken with the required minimum of rigour, care, and honesty. More broadly, three types of problems emerge: a lack of consultation, of informed consent and a risk of abuse of trust.

#### 3.3.1 Lack of co-designing

Employees are often not properly included in co-designing and decision processes ahead of the deployment of the devices. Moreover, fairness issues may arise if a consultation procedure only involves a subsection of representative employees. Such situations fail to respect the value of workers’ point of view and their autonomy ^(7,9,17,19,21,26,28,30,41,42,48,50,53,59)^. Workers have insider knowledge about what could be improved and their security needs; their participation in the developmental stage of technology can also help design more efficient tools. If not consulted, workers may be provided with IoTs they do not want and resist using, or with IoTs that fail to target the most important work security issues, or whose default settings cannot be adapted in ways that make the outcome decisions of the system meaningful ^(2,21,30,42)^. Moreover, employees may have different sensibilities about data security issues. For instance, they may disagree fundamentally with IoTs collecting sensitive data about them, or they may have context-specific reactions (e.g., allow devices to provide relevant information to health providers or to national health authorities, but not if their employer can access them). This context sensitivity tends to be overlooked in the rush to develop and deploy technical solutions for good OSH purposes ^(17,25,38,39,58)^.

Nevertheless, it may ironically happen that employees demand the deployment of the technology and agree to the implicit constraints and risks to which they then will be subjected. In such situations, the ethical issues are co-generated by the victims, and the difficulty is to protect employees against their judgement and decisions ^(26,58)^.

#### 3.3.2 Lack of informed and free consent

When employers plan to use IoTs, they are expected to obtain employees’ consent. Nevertheless, *informed* and *free* consent is seldomly obtained. Often, workers tend to be insufficiently informed, or transparency is lacking about what will be collected, from whom, for what aim and for whom’s profit ^(1,3,12)^. In other cases, the relevant information is transmitted in an unfriendly or non-understandable way. Users (especially those with little literacy or ease with the technology) are likely to be unfairly overwhelmed by the burden of gathering and understanding all relevant issues, a task requiring skills that cannot reasonably be expected from users ^(1,10,19,28,29,37-39,41,42,47,54,55,59-61)^. Workers rely heavily on employers’ willingness to consult them and to provide them with relevant information and choice options. High-quality consent often involves complex procedures and explicit responsibility taken on the part of employers, which they may be reluctant to engage in. Sometimes employers have strong incentives, such as industrial secrecy, for retaining part of the relevant information ^(4,7,16,17,19,39,52,54,55,59-61)^.

Furthermore, the line between voluntary and compulsory consent is not always clear-cut. For instance, due to their contractual relationship, power asymmetry, peer pressure, or lack of the privacy prerequisite for an autonomous decision, employees may not be free to refuse or negotiate. If they do so, they may face negative consequences such as being stigmatised or marginalised by employers or peer workers, or excluded from goods or services that rely on the collected data or on the use of the device. In general, it is more difficult to reject practices that are already largely accepted among workers ^(3,8,13-15,17,29,30,40,41,46,58)^. Moreover, asking for free informed consent from a population that is vulnerable in many respects (e.g., less educated, from a different social background, with precarious health), and not necessarily aware of the importance of consenting, requires pro-activity and communication skills that employers and occupational health staff seldomly master ^(10,54,55,59)^. Consequently, in many situations, the consent obtained is of poor quality (e.g., based on external pressure, biased presentation of information, misleading short-term benefits) and sometimes partly fabricated ^(3,15,18,25,32,39,41,46,53,56,60)^. In some cases, deployment of devices or data collection is practised without any form of consent. For instance, this is the case with devices that capture data in private contexts where family members are interacting with the employees. It is also the case with devices that are widely distributed or that operate over long periods of time, one may forget to ask consent. In the latter case, for instance, periodic consent renewal is rarely done, although it is important to remind users of the ongoing monitoring ^(7,43,61)^.

In the case of data reused by third parties in aggregated form for external purposes such as medical research, device optimization, or public health goals, consent procedures are even more difficult to follow and often poorly practised. For instance, it remains challenging to provide useful information about possible linkage and recoupling of data by third parties, or about the risks of disclosing sensitive information about specific social groups ^(46,54,59)^.

Finally, deployment of technologies that have been insufficiently consented to can elicit practical difficulties such as poor adherence or acceptance of the device from workers. It also raises major ethical issues such as disrespect of workers’ autonomy, and all sorts of sweeping consequences that workers would have resisted if they were informed ^(5,20,32,40)^. In a worst-case scenario, if, for some reason, employees fail to use an IoT properly, this could lead to the collection of bad quality data. Unknown to the employees, these data may then be used to train models (deemed to become suboptimal) or to make wrong predictions about employees’ health status. This in turn may lead to suboptimal or problematic managerial decisions. Without proper information and consent procedures, employees are oblivious of the causal role of their original failure and have no way of avoiding such detrimental causal chains ^(23,24,46)^.

#### 3.3.3 Abuse of trust

The literature highlights a third type of problem regarding workers: the risk of abuse of trust. In some cases, workers may place unconsidered trust in their employer or in the health specialists within the company and, therefore, may not objectively balance risks and benefits while being consulted about the device. This lack of critical view is a sign of vulnerability which leaves room for possible abuse ^(3,5,17,49,59)^. In other cases, workers may not be conscious of the extent of surveillance or dual-use practices because they are not transparently informed and the device is invisible or seems (deceptively) harmless ^(28,38,56,60,61)^. The discovery of such abusive secondary uses will, of course, be detrimental to trust relationships. When deploying IoTs, an additional task for companies to fulfil is to help maintain trust relationships with employees ^(33,53)^.

### 3.4 Unintended and unpredictable adverse effects

A third and large set of ethical issues is related to the adverse effects that can occur with the deployment of IoTs for OSH purposes. Their potential autonomous continuous learning processes and prolonged use may generate major structural and cultural changes within organisations and beyond. We identify no less than 14 adverse effects in the literature, and the authors elaborate on an important list of issues related to unintended and (partly) unpredictable short-or long-term adverse effects of deploying IoTs. They highlight that these effects are not given sufficient consideration and often become apparent during late testing phases or after the deployment of the technology, by which time it is too late to make changes.

#### 3.4.1 Negative impacts on employees’ physical and mental health

The feeling of being constantly monitored by invasive tracking systems that blur the limits between professional and private life may, in the long run, generate feelings of being under continuous threat, significant fatigue, stress and anxiety. These states of mind are conducive to burnout ^(4,9,14-16,25,26,34,44,51,56)^. The deployment of IoTs may also involve additional tasks related to the device or organisational changes that further destabilise employees or generate fear ^(15,29,33)^. Moreover, devices developed for creating self-tracking habits can generate cognitive overload, headaches, some forms of cyber-sickness, health-related anxiety, an excessive and damaging obsession with personal health, techno-stress, or addictive self-tracking among users ^(1,5,13,15,19,24,26,28-30,34,37,38,42-45,50,53,56-58,61)^. Even if devices may be helpful for reducing some risks, when co-deployed with a demand for an intensification of work, this effect can counterbalance any safety and health benefits ^(9,16,51)^.

In case of poor design, IoTs may provide wrong or biased information or directives or generate a limited and poor view of broader social and contextual factors that are important for safety and health. They may be burdensome to use, especially for some groups of workers, such as elder workers. They may be badly secured and create new unintended health or safety workplace hazards, such as mechanical, electrical, thermal, or chemical risks. These issues are particularly problematic when the implemented devices have little or marginal demonstrated health-related benefits, or when they are introduced as a replacement to traditional and more effective OSH methods ^(2,3,6,17,19,21,28,30,32,34,35,43,44,46,50,51,56,59)^.

#### 3.4.2 Weakening of workers’ power, competencies and self-confidence

Another adverse effect is that workers may progressively lose some competencies (e.g., despecialisation, decrease of critical thinking or risk alertness) because the devices take care of some of their previous duties. In the long run, workers who overly rely on technology for assessing health and security risks may become less skilled at identifying these risks ^(7,9,21,34)^. Conversely, users may become unsure about their own perceptions of judgement when the devices produce outputs in contradiction with their evaluation (e.g., about current stress level or about what should be done), or they may not feel entitled to rely on their evaluation when the device fails to spot a risk that they have seen. As pointed out by Mc Aleenan^(30)^ *“there is a potential for a taught helplessness syndrome emerging from this type of technology where workers rely on it to inform them rather than on their own observations and judgement. Conversely if the worker, in the absence of an alert relied on direct observation to stop work, how would this be interpreted by management.”* Some authors even argue that prolonged use of IoTs that track and send feedback on a reduced set of personal features may generate a reductionist understanding of health and selfhood and profoundly alienate users from their true selves ^(1,16,19,29,46)^. Moreover, the devices may require workers to acquire additional technical skills that they struggle to master ^(10,18,19,43,51)^. Overall, the prolonged interaction with the devices may affect not only employees’ agency, workplace role, and competencies but also their self-image and self-esteem, and generate feelings of frustration, of being disrespected, of inferiority and subordination to machines, and possibly feelings of incompetence and fear of job loss ^(13,18,21,26,28,30,31,37,42,44,47,56)^.

#### 3.4.3 Negative impacts on working processes and habits

An IoT can also be a cause of distraction from work if it generates over-dependency on self-tracking or if it triggers unproductive and disruptive competition between workers ^(1,13,20,21,24,26,28,29,37,38,42,56)^. It can alter working practices if employees are aware of being observed or because they are incapable of adapting to new workflows ^(9,16)^. Backfiring effects such as reward hacking or new rule-breaking behaviour may occur. For example, employees may develop new behavioural strategies to circumvent the obtrusiveness of an IoT or to neutralise an employer’s excessive collection of personal information; thereby they may take more safety risks or increase communication barriers in the workplace ^(5,6,20,21,44,49)^. In the opposite direction, the ubiquity of the system can lead workers to set excessive constraints on themselves. For instance, they may be so constantly responsive to the inputs of devices that they work to exhaustion ^(26)^. Moreover, when IoTs fulfil tasks that were previously completed by workers, they can unsettle employees and working routines by challenging conventional knowledge and expertise ^(4,7,14,20,40,42,44,61)^.

#### 3.4.4 Risks of breaches of sensitive information

Another issue may arise when managers, co-workers, or external third parties (individuals, companies, State) acquire knowledge of private information about workers that was previously hidden ^(6,21)^. Massively collecting and storing sensitive data increases the risks of data theft, data leaks, or unwarranted disclosure of personal information. Information breaches can happen in non-ordinary situations such as cases of cyber-espionage, but also in ordinary working contexts such as when security alerts targeted at one worker are heard or visible to other workers ^(22,25,43,48,51,53,58)^. These risks are more likely to occur when workers’ data sovereignty (i.e., right to access and to exert control over their personal data) is not guaranteed or made reasonably accessible because workers do not use the device to protect themselves against malicious use, if the devices used are not technically robust against hacking, or if the data transmission and storage process is not managed in a secured manner ^(3,21,25,40,41,45,59)^. One can easily lose track of the life cycle (i.e., where and for how long are the data accessible or transmitted to whom) of data that can be copied and stored ^(45,52,61)^. In some cases, information breaches can even be a risk for the company itself, such as when strategically relevant internal deficiencies are disclosed to competitive external actors ^(17)^.

#### 3.4.5 Pressure for normalization and exacerbation of social stigma

Since IoTs allow for large-scale quantification and comparisons between workers, new expectations or social norms at work oriented towards healthiness and effectiveness may be shaped. The phenomena of co-surveillance, inappropriate competition between workers, or social pressure towards healthy behaviour or towards presenting oneself as being healthy may arise ^(7,13,30,41)^. Such trends of normalised expectations at work usually operate at the expense of individuality and individual differences ^(7,21)^. This can have negative impacts on workers, especially those who are already fragile or who do not conform to or meet the normative criteria induced by the health-promoting technology. For instance, exaggerated fitness goals generate health risks and social shaming of those who do not meet the standard ^(8,10,13,16,32,37,41,45,46,49,59)^. More generally, a large deployment of IoTs may create pressure for conformism and normalised behaviour, with its correlated effects: loss of individual initiative, self-contained or innovative decisions, despecialisation of workers’ job duties and activities, decrease in workers’ acuity in evaluating OSH risks, and blurring of individual responsibility in case of hazard ^(1,2,7,28-30,40,42,46,56)^.

#### 3.4.6 New or increased existing inequalities

Workers may also experience various forms of discrimination, stigmatisation or exclusion (by employers or colleagues, in the workplace or above). It can be the case because IoTs disclosed information about workers’ health risks, or due to workers’ refusal to use the proposed device, or their difficulty conforming to normalised expectations generated by the devices, or the overtaking of some of their tasks and competencies by the technology, or on their lack of ease with the technology ^(1,3,7,12,15,18,29,42-46,48,52,57)^. Regarding the latter issue, the useful notion of “digital divide” and its overlap with existing vulnerabilities is also discussed ^(34,48,51)^. In all these instances, some categories of workers will be more at risk of suffering in one way or another because they feel insecure, stigmatised, unequally treated, experience true discrimination, etc. Older generations of workers, workers who are more likely to develop work-related illnesses, or minority ethnic groups will be particularly impacted by the unequal distribution of benefits and hazards of the deployed technology: IoTs may affect differently or under-perform on these groups of workers because they do not fit with the “ideal worker” in light of which the IoT is configured, or because they do not master the technology (due to literacy or language barriers), or because the devices’ output reveals “unliked” sociocultural features of workers, creating avenues for a series of unequal treatments (income gaps, unfair work-tasks distribution, etc.) ^(1,10,15,21,27,29,43,44,48,59)^. In this context, some authors discuss the notion of “occupational health inequity,” referring to “*avoidable differences in work-related fatalities, injuries, and illnesses closely linked with social, economic, and/or environmental disadvantages*” ^(34)^. Despite OSH goals, the right to fair working conditions may be insidiously endangered for some categories of workers ^(19)^.

#### 3.4.7 Weakening of human relationships at work

If IoTs replace humans in security and health prevention messaging, they will modify social interactions and communication paths, or teamwork in the workplace. Such a situation may generate communication errors or break important relational ties ^(9,13,18,28,42,56)^. To illustrate, an automated system may create unforeseen communication issues by providing incentivising messages that are difficult to understand by end-users, thereby generating stress and mistrust among employees ^(44,45,59)^. The prolonged use of devices designed to avoid risky human interactions at work (e.g., a contact tracing app during the Covid-19 pandemic) may contribute to social isolation, social anxiety, and hostility among workers ^(16)^. Improved safety measures with IoTs that enable an intensification of the workload may also decrease social relationships. In this context, employees may be unequally willing to endorse and able to engage with the technology, and some may feel abandoned or dehumanised ^(24,28,30,34,37,44,56)^. Overall, turning some OSH tasks over to IoTs may weaken communication, understanding, and compassion between employees and occupational physicians or health and safety managers. In some cases, workers may even end up creating inappropriate ties with the technology, by attributing intelligence, empathy, or trustfulness to an AI system ^(42)^.

#### 3.4.8 Weakening of trust relationships at work

Even when IoTs are deployed for addressing important health and safety issues, the above-mentioned feeling of being constantly monitored, evaluated and possibly discriminated, and the weakening of human relationships may endanger trust relationships between employees and managers, occupational physicians or OHS managers, and weaken safety and health management and culture. Mistrust and a correlated reluctance to use IoTs is most likely in case of abusive surveillance, when the outputs of the devices are based on opaque algorithms, when the deployment of IoTs is not properly explained and accompanied by transparent information about the reasons for the deployment, the associated risks and alleviation measures, or when workers have not provided explicit consent ^(1,4,5,7,20,30,32,35,38,40-42,45,49,51,53,59)^.

#### 3.4.9 De-responsibilisation of employers

Considering that IoTs are generally embedded in AI systems that can make assisted or autonomous decisions, part of OSH responsibility may be out-or subcontracted to the machine and thereby shifted outside existing managerial or company protocols. This raises all sorts of understanding and regulating difficulties. For instance, it may blur the channels of internal accountability in case of adverse events or professional illnesses, and lead to a back-scaling of employers’ OSH legal obligations ^(42,44)^.

Moreover, as they have done something by deploying IoTs for OSH purposes, employers may feel discharged from the duty of implementing further prevention measures or healthcare services ^(15,16,29)^. While discussing these issues, some authors point out the risk of over-responsibilising employees for their security at work or for their health status, since they have received OSH counselling and inputs by the devices ^(8,12,14,31,45,46,53)^. The increasing deployment of IoTs for health purposes in working and in private contexts is accompanied by sweeping expectations that workers (and citizens) pay an active role in self-tracking their health and safety risks, and in caring for themselves. They thereby become responsible for managing those risks. If they fail in this task despite the support provided by the technology, they tend to be held responsible for the hazards and illnesses that plague them. Such a trend, which is observable not only in working contexts but in society at large, is correlated with a de-responsabilisation of employers and public health authorities, and with a decrease in funding for professional social support and healthcare services ^(1,10,29,46,59)^.

#### 3.4.10 Normalisation of surveillance practices

In the long run, surveillance may become the default norm rather than a measure that needs to be justified. Such a change of paradigm may occur gradually without stakeholders noticing. As surveillance practices become more commonly used in a variety of contexts (home office, business travels, etc.), workers may become less aware of the obvious associated risks, especially in cases of passive monitoring (no particular action needed from users) where workers are not regularly made aware of the fact that they are monitored or in the context of gamification practices when individual progresses can be scrutinised by “followers” or compared against others within seemingly harmless challenges or competitions ^(3,12,15,17,24,29,42,46)^. This can gradually lead to acceptance of more exacerbated or invasive forms of surveillance (e.g., facial recognition, multiple data collected on the same individual, etc.), possibly up to the “internet of bodies” with devices inserted into humans, and to a society where everything is connected through an “Internet of Everything” ^(43,46,59)^. Such forms of intensive monitoring are usually of interest to companies that commercialise or deploy the devices at the expense of workers ^(32,56,59)^.

#### 3.4.11 Dystopian and dehumanized workplaces

IoTs are also portrayed as potentially contributing to the emergence of dystopian scenarios where a great range of decision-making becomes automated and where employees are submitted to excessive algorithmic management, meaning that the workload attribution, time management, or short-term on-demand employment of vulnerable workers is managed by automated AI systems. A large deployment of IoTs may blur the boundaries between AI and humans, raising the question whether standards originally designed for ordinary human workers still hold ^(15,18,42,44,51)^. The risks of dehumanising the workplace and its negative repercussions are abundantly discussed by some authors ^(7,15,18,42,44,56)^. Workers may lose their individuality, autonomy, expertise and value within the company and become opportunistically utilised as “*farmed and domesticated entities (..) rather than autonomously involved in authentic health maintenance initiatives*” ^(32)^. If workers have little choice but to accept workplace monitoring despite its constraining and dehumanising effect, it may stifle their motivation and creativity while fostering suspicious beliefs ^(14,30)^.

#### 3.4.12 Increased controversies surrounding IoTs and AI in public discourses

As we have seen, IoTs for OSH purposes may contribute to the general trend of workplace surveillance and generate numerous adverse effects. Workers and citizens are likely to become aware of these ethical issues, as is the case when media disclose how targeted advertisements stem from massive repurposing of data collected by IoTs. Such awareness often does not facilitate public discourse on tracking devices, and on AI in general. It can generate strong controversies conducive to a polarised society ^(19,32)^. Some authors regret the lack of transparent information about flaws in the technology and in its underlying algorithms, arguing that it is an obstruction to productive public discussions on how AI systems can be improved. Mistrust towards technology and AI is also problematic if it hinders the deployment of technologies that permit socially valuable advancements ^(18,35,44)^.

#### 3.4.13 Disruption drivers of public health, social values, and employment policies

In the long run, the widespread deployment of IoTs for OSH purposes may also have a wider impact on the social understanding of health and safety issues. IoTs enable efficient assessments, predictions, and behavioural recommendations without imposing solutions. Stakeholders are mostly responsible for following (or not) the automated injunctions made by the devices. In this sense, extensive use of such technology reinforces the neoliberal understanding of social healthcare duties: the domain of OSH tends to be reduced to prediction and information tasks, leaving employees the responsibility of taking care of their health capital. The rhetoric of users’ empowerment is put forward while promoting IoTs, leaving in the shadows the employers’ and state’s duties to provide social healthcare to ill workers and citizens ^(2,3,8,9,12,15,16,29,46)^.

In parallel, health data philanthropy is promoted as a means to promote medicine and healthcare. Related to this trend, one author worries about a correlated depreciation of the value of privacy, depicted as individualistic and as an opposite of openness, transparency, and public good. Employees are increasingly expected to contribute to the common good by taking care of their health and by giving their private and sensitive data ^(12)^.

A further issue is the gradual increase in importance of private health industry. OSH is one place among others in which private companies developing health-related devices colonialise the public health domain, thereby disrupting traditional public tasks and responsibilities ^(7)^.

Similarly, IoTs for OSH purposes contribute to the enforcement of neo-Taylorism practices in the workplace. Employees are exposed to daily automated injunctions are quantified and assessed in multiple ways by AI systems and are sometimes favoured (or disfavoured) based on those assessments. Such work organisation compromises workers’ individuality and capacity to organise and form resistance to these new management tools and contributes to the social divide between those who have power and those who are reduced to quantified workers ^(1,14,16,21)^. These practices may have a widespread impact at the societal and policy level, notably by weakening the social aspect of employment policies ^(1,14,16,29,43,46,51,55)^.

#### 3.4.14 Negative environmental impact

Last identified adverse effect is the significant environmental impact (carbon footprint, environmental consequences of the extraction of rare material, recycling issues) of such connected technology, which becomes a serious issue in time of global climate change and environmental insecurity ^(9,14,31,56)^.

### 3.5 Suboptimal data management

We identified another group of five ethical issues in the scholarship. They concern suboptimal data acquisition, transfer, storage, processing, and dissemination. They are linked to situations already mentioned and are particularly likely to occur in the case of IoTs that collect sensitive health information and may be deployed at workers’ homes in the context of a home office.

#### 3.5.1 More data than needed are collected or stored in the long term

First, there is the temptation with IoTs to collect more data than needed for OSH purposes. For instance, additional data may be collected for the purpose of personalising the outputs of the systems. Sometimes, additional data are collected without clear goals, just because the default setting of the system allows it, or just in case these data could be relevant later. Collecting a wide variety of data on the same individuals during a continuous period of time in different contexts (work and home) are factors that increase the risks of deanonymisation of sensitive data at the expense of equal treatment. Problematic situations also occur when data are collected at a time for a particular purpose, but stored longer than needed, thereby increasing the risk of later reuse. This was an issue at the end of the COVID-19 crisis ^(39)^. Once a worker’s profile has been created in a database, it can “persist” endlessly. These are all cases of breach of the principle of data minimization and are conducive to numerous ethical issues such as excessive surveillance, increased mistrust in workplaces, etc. ^(5,12,18-20,32,38,39,42,49,52,59-61)^.

#### 3.5.2 A too-large spectrum of stakeholders has access to the data

Second, depending on the device’s purpose, various actors may have access to the data, such as the IT unit and services, managers, co-workers, OSH staff, third-party medical institutions, network infrastructure suppliers, government bodies, etc. It may be unclear (or insufficiently managed and controlled) where to set a limit on who is authorised to access what data, for what purpose and until when. For instance, it is often unclear why human resources should receive health data collected for OSH purposes ^(12,17,25,45,49,51,53-55,59)^. In numerous situations, third-party companies are also given the task of storing or processing the data (e.g., conducting the data analysis). These companies may insidiously resell part of the data (e.g., to private health companies interested in the health profile of future clients) or use them for other purposes (e.g., for sending targeted ads to users) ^(1,13,41,53,56,59)^.

#### 3.5.3 Lack of secure systems and procedures

Next, IoTs may not be robust enough to react against unauthorised data access by third parties, cyber-attacks, or malicious uses, either due to lack of inbuilt security measures, poor conception, or to failed maintenance. The collected data may be insufficiently de-anonymised, allowing breaches in personal privacy when re-identification (usually with correlative analysis on different datasets) is operated by a third party ^(1,3,5,12,17,18,22-24,27,28,31,35,37,38,42,43,48,54-56,58,60,61)^.

#### 3.5.4 Low quality of data or of data analysis

Moreover, imprecise or partly irrelevant data, as well as the use of suboptimal or biased algorithms, may lead to unnuanced or incorrect interpretations. For instance, this may happen if the data used for training the system are not representative of the target OSH domain in the workplace or if the technical system is unable to represent the diversity of workers and their characteristics in an appropriate format. When those limitations are overlooked, inappropriate or discriminatory outputs and decisions may occur or be reinforced ^(2,3,7,9,14,15,17-19,23,25,32,34,35,41,42,44,46,48,49,51,52,55,59)^.

#### 3.5.5 Numerous devices deployed in the workplace

Finally, if an expanding number of devices is deployed, each with its own purpose, technical specificity and database, it becomes difficult to ensure a centralised governance and effective security controls ^(39,61)^. It may be a further cause of distress for employees who do not feel at ease with the trend towards technological advancement ^(18)^.

### 3.6 Contextual factors that are conducive to ethical issues

Contextual factors compose the last major category of topics discussed in the literature. Even though they are not ethical issues in themselves, specific contextual factors may be conducive to ethical issues.

#### 3.6.1 Blurred limits between professional and private life

If used in the context of a home office or on portable devices such as smartwatches, IoTs contribute to blur the limits between professional and private life, especially when surveillance practices extend over strict working activities and when they operate in the background, more or less outside user awareness. This reality induces organisational difficulties (e.g., how to set clear boundaries between working and free time) or increased stress levels among workers who may feel constantly monitored in their daily routine and struggle to take breaks from work. It may also generate a lack of clarity about employers’ rights and duties ^(4,5,7-9,12,17,21,24,26,34,41,42,45,46,55-57)^.

#### 3.6.2 Techno-optimism and lack of ethical awareness among stakeholders

Stakeholders may lack critical thinking. Developers of IoT and AI systems are not always clear about the spectrum of possible wrong uses or problematic motives for using their products. Employers deploying the solution, may be overenthusiastic and readily assume that the technology is objective and efficient, that it will benefit the workforce, without foreseeing its limitations or being aware of the risks of lack of efficacy, data re-purposing by third parties involved, etc. For instance, employers readily accept methodologically limited whitepapers produced by the companies that commercialise the IoTs, and if at all, the efficacy of the devices is usually examined after their commercialisation and deployment in the workplace ^(15,19,21,22,24,46,51)^.

If relevant stakeholders show such overconfidence in technological solutions, combined with low ethical awareness, one can expect little vigilance and motivation to measure appropriate outcomes, no systematic risk assessments of all process stages, and a lack of mitigation measures. Stakeholders may be satisfied with superficial checks for legal acceptability. They may systematically underestimate foreseeable risks and associated harms and overlook the ambivalence of their use of the technology when it implies some form of surveillance ^(26,30,42,46,59)^. For instance, when IoTs are used to assess employees’ health conditions, only a narrow range of easily measurable or quantifiable criteria can be taken into account, leaving out relevant contextual factors such as individual or socio-cultural characteristics. If such limitation is not taken seriously due to an over-optimistic appreciation of the device, biased or suboptimal OSH measures may be taken by OSH staff and occupational physicians. Consider the further illustration of an IoT provided to cleaning ladies in the hotel industry to assist them in case of sexual assault. In that case, location tracking capabilities of the device need to be particularly developed to quickly find the worker and send assistance: efficacy is correlated to a high degree of surveillance, and employers are likely to undermine one or the other difficulty ^(17,19,25,55)^.

Overall, human-centred policies and ethics-by-design procedures have not yet become standard or harmonised. This absence can amplify the adverse impact that IoTs have on employees. Authors also worry that stakeholders may have to deal with too rigid and fixed ethical frameworks, which make them ill-equipped to deal with the novel demands of a rapidly evolving technology ^(19,41,42,44,51,53)^.

#### 3.6.3 Emergency situations

Emergency situations are a contextual factor that can bear up the deployment of IoTs. One known example is the contact tracing apps during the COVID-19 crisis, which were strongly endorsed by employers to reduce the propagation of the virus. In such a situation, there is less time, funding, and mental availability to evaluate the appropriateness of the purposes and to consider the long-term adverse effects of a technology. Once it is deployed, there is a risk of keeping the solution in the long run without a proper re-evaluation procedure or despite the counterevidence of its proportionality ^(38,39,45)^.

#### 3.6.4 Complexity and lack of competencies

The fast-evolving field of technology means that the developer’s community may lack consensus on how to use, integrate or optimise the technology and at the end of the chain, users may be insufficiently informed ^(1,37,43-45,56)^. A myriad of devices are developed at a fast pace and deployed for a large variety of applications. From the development to their implementation, numerous actors, decisions, and information transfers are involved. Each can be limited in many ways. For instance, some IoTs allow for automated decision procedures based on systems containing self-learning algorithms. Consequently, they raise explainability issues: even designers of the devices do not have an operational understanding of how the devices make predictions or recommendations^(22,48,51,60)^. Some systems are grounded on strong theoretical assumptions that are not clearly spelled out or justified: this is typically the case for devices supposed to measure well-being, a state that can be defined in many ways and tracked with a large variety of observable features^(19)^. Operational conflicts may occur at the deployment stage of the IoT: since developers do not use common languages, operating systems, and evaluation practices, compatibility issues with other technologies used in the same workplace are likely to emerge^(37)^.

During the implementation stage, actors involved (medical staff, OSH team, workers, public health decision-makers) may lack proper training and education ^(9,22,24,29,43,55)^. Moreover, once data are collected, given the number of stakeholders involved, it remains particularly challenging to keep a reasonable control on the flow and use of data ^(19,61)^. Overall, when new IoTs are deployed for the first time, it is difficult to foresee all possible hazards. To some extent, it is a form of unregulated social experimentation^(14,22,26,28,42,43)^.

The complexity of the technology and data process makes it particularly difficult to provide clear and comprehensive information allowing stakeholders to anticipate how workers may be impacted by the deployed IoT. It becomes more challenging to respect workers’ rights over their data^(9,24,28,48)^. This is particularly true when workers lack the digital literacy and competency to use and understand the technology, or when the devices are regularly updated, iteratively developed, redesigned and extended, or when they include opaque machine-learning algorithms that cannot be traced or controlled by humans^(10,28,30,37,42,43,45,52)^.

Such complexity generates new illiteracy and its correlated risks. The difficulties involved in understanding, explaining, and evaluating IoTs and AI systems and their outputs are obstacles to stakeholders’ (especially workers’) trust in the deployed technology and their capacity to manage it. Some stakeholders may not even try to gain minimal technical knowledge, which does not help informed decision-making^(1,2,22,24,35,43,47,52,53)^.

#### 3.6.5 Lack of scientific evidence or peer-reviewed data

In many cases, there is a lack of high-quality scientific evidence (e.g., based on randomised control tests and study designs with high external validity) that the devices are valuable tools for achieving their goals (taking into account long-term efficacy and adverse effects) in the particular workplaces, compared to more human solutions. This makes it difficult to evaluate whether it is proportionate to implement them in the first place or to keep using them once deployed^(2,3,6,25,32,35,37,41,42,44,45,51,53)^. For instance, not enough (or not clear enough) measures are collected, leaving too much space for interpretation, and making it difficult to evaluate whether a device positively contributes to work health or safety^(2,56)^. Interestingly, the difficulty of accessing high-quality data is sometimes caused by legitimate privacy protection measures (data anonymization, limited access to sensitive data) set up to prevent opportunities for abuse^(1,40)^. Or it may be due to market constraints: companies are reluctant to disclose the parameters of their algorithms and to provide insight into the data used to train their AI systems because private investments are at stake^(44)^.

A connected topic is the difficulty of evaluating the accuracy of the outputs produced by theory-based algorithms trained on selected datasets that are not always close to the workplace context or to the typology of workers who will use them. Deep-learning systems are even more difficult to evaluate since they work in a non-transparent manner ^(1,3,37,58,59)^.

#### 3.6.6 Costs of ethical safeguards

The deployment of IoTs that fulfil ethical standards often involves huge additional time and costs: investment in the development of more secure systems (devices, software, etc.) which require more technological expertise; development of more complex implementation strategies that take care of workers’ vulnerabilities; organisation of proper information and training for managers and users; ensuring long-term maintenance and security monitoring, etc. It is unclear who is willing and who should pay for these costs (developer companies or companies who buy the devices), especially in the context of a competitive market. Strong conflicts of interest are at stake: for instance, when monetary incentives are involved, it is difficult to produce an objective cost–benefit analysis of security measures. Moreover, if by some mechanism, strong security measures are imposed on companies (for justified ethical reasons), unfair market advantages may arise, since the costs (especially high when dealing with AI or interconnected systems) may not be affordable for the public sector or small-to-medium-size companies in contrast to big and wealthy firms ^(1,2,29,31,35,38,40,43,47,50,55)^.

#### 3.6.7 Lack of clear overall governance

Worries are expressed regarding the lack of specific regulation framing the deployment of such systems, at national and international levels, and regarding the fact that existing legal frameworks that may be applied vary greatly by country. Notably, authors worry about how one should apply data privacy regulation (GDPR), medical device regulation, labour law or contract law to the deployment of IoTs, while taking into account all country-and region-specific challenges ^(9,12,15,23,24,35,39,41-44,48,54,59,60)^. Authors also worry about the extent to which companies and third parties that have some access to sensitive data comply with regulations and enforce robust privacy practices ^(41,42,48,53)^. A meaningful application of existing laws is particularly challenging due to the complexity of the interconnected technology and insufficient legal and technical literacy among policy and company leaders^(1,9,44,60)^.

The lack of standards, guidelines, institutional safeguards, and regulatory bodies is also an issue. Rights and ethical principles are sometimes too abstract and not amenable. International and national laws are maturing at a slow pace compared to technological innovations. Even when clear and applicable laws and guidelines exist, they remain ineffective if they are not sufficiently well communicated and enforced by regulatory procedures and institutional bodies ^(3,9,12,22,25,42,44,45,48,51,59)^. Within the private sector, it is also difficult to find appropriate institutional procedures (e.g., validation processes, regular staff training) or departments that oversee and regulate the use of IoTs by conducting controls and audits, and by delivering authorizations based on strong security controls. There is also a lack of specific protections for vulnerable groups of workers, such as older populations. Consequently, those who develop or deploy the technology in the private sector may not comply with law because of ignorance (or absence) of guidelines and policies, lack of common standards (exemplar methods and practices), absence of strategic frameworks for the development, acquisition and use of IoTs, and overall absence of regulatory control ^(1,12,19,29,37,42-45,48,50,55,59,60)^.

Without clear and standardised ethical guidelines, laws, independent regulatory bodies or related agencies, it remains unclear what is expected from developers, deployers, and users of the technology. For instance, to what minimal standards are they expected to abide? To what extent are they expected to engage with contextual trade-offs and interests, such as protecting the privacy of specific vulnerable groups at some additional cost or at the expense of organisational efficacy^(15,17,59)^. It is also unclear who is accountable at each step of the process in case of security or health hazards due to malfunction, error, lack of integrity, or in case of unequal treatment of workers^(9,17,18,28,31,37-39,42-44,51,56,60)^. One illustration is the fact that gig economy workers are less protected by law due to the lack of clarity on their working status^(34,59)^. Another illustration is the case of IoTs deployed with insufficient procedures to handle cases of conflicting evaluations prompted by the device and made by employees, making it difficult to define employees’ rights (to contest the systems’ outputs) and responsibilities^(42,44,48)^. Without clear regulation and governance, it will also be difficult for workers to know what are their rights (e.g., do they have property over their health data? who else has it?), how these rights are respected by the company (e.g., who has access and possibly owns their data?), whether their rights are protected by the administration, and how they can fight for them^(1,3,5,12,32,37,38,45,48,55,56,61)^. While discussing these issues, some authors regret the current neoliberal political trend tending to less regulation and more individual responsibility on the shoulders of employees^(29)^.

Lack of guidance or constraints enables the flourishing of a competitive market of self-tracking health devices, with products selected for their economic viability rather than ethical acceptability. These difficulties increase if the health care system is not clearly structured and too many companies or individuals can claim some right of access to workers’ health data, or in the case of strong business interests (e.g., needs for data collection in a competitive market), or if the pace of the regulatory process is not adapted to the dynamic and short life cycles of technology deployment^(29,32,44,49,55)^.

Effective regulation, standards, and governance is made difficult due to a number of reasons: the inherent difficulty of conflicting rights and constraints (e.g., right of workers’ privacy and information, right of industrial secrecy, necessity of business productivity, etc.), the lack of human oversight on what is deployed, the technical complexity of the technology and the difficulty to ensure the interconnectivity between the different systems (e.g., large number of protocols and devices that are deployed across different application domains that are more or less interoperable with new and old systems, and that generate various types of data), the lack of expertise among stakeholders, and the lack of clear task and responsibility attribution to those stakeholders ^(2,15,24,26,31,42,43,48,55)^.

## 4 Limitations

Since very few articles that we found explicitly focused on ethical issues *of IoTs for OSH purposes*, we often had to apply relevant general theoretical points made by authors to our study case or adapt points originally made with a different focus. However, we do not think that by doing so we distorted the original authors’ points of view. In most cases, the ethical concerns the authors raised apply to a variety of cases, including IoTs for OSH purposes.

Due to paywall constraints, we did not have access to 8 potentially interesting articles. However, since we approached saturation of content after reading 40–45, it is unlikely that we missed important contributions.

This review does not include grey literature in which additional ethical issues may be discussed. For instance, one interesting highlight that came up during the review process of this publication is the ironic safety risks posed by IoTs for OSH purposes: these devices often involve AI solutions that are limited regarding their explainability, analysability, and relevance of output data. “Black boxes” and limited datasets may generate safety risks or poor health & security standards. Safety monitoring of IoTs for OSH purposes is therefore required (44, 69).

Since the topics presented here overlap and intersect in myriad ways, they could have been organised differently. This methodological difficulty is unavoidable and has already been highlighted by others (21).

## 5 Discussion

The list of ethical issues resulting from this literature review is impressively (and unexpectedly) furnished, varied, and substantial. Many issues would deserve an extensive discussion that we cannot make in this paper. Some of them echo well-known topics which have also been highlighted in broad-scoping theoretical works [e.g., (21, 39, 48, 63, 64, 70–72)]. Nevertheless, none of these studies focussed on the impacts of IoTs primarily deployed for good purposes such as OSH. This indicates that, these devices are generally not understood to be ethically problematic since they are deployed for noble goals (the health and security of employees) and fit within the new trend of the 5.0 industry that places the well-being of workers at the centre of production processes (73). As a result, they tend to escape the necessary ethical scrutiny (51). The blind spot in the literature and among policymakers is appealing at a time of narrative in favour of technology (e.g., AI for good, data for good). To a certain extent, it also illustrates the framing power of academic and political discourses.

Moreover, among the five categories of issues that we point out, three are focused on direct and tangible elements: the employer, the worker, and his or her data. They refer to easily observable and abundantly discussed difficulties (e.g., lack of consent, surveillance, exploitation, data security) which we expected ahead of conducting the review. We were however more surprised by the two further categories that cover adverse effects and contextual factors. These categories are surprisingly furnished and reveal a deeper layer of complex and interconnected ethical issues that were more seldomly addressed. Our scoping review method helped to reveal these topics discussed in different disciplines (sociology, business ethics, AI ethics) that do not systematically communicate with each other. For instance, some authors in the social sciences literature have already made calls for societal impact assessments, but their concerns did not bridge to other academic fields. In this context, we believe that a robust cross-disciplinary ethical analysis can play a role in better informing IT developers and OSH policymakers and practitioners.

Facing the wide spectrum of ethical issues, and in particular the imposing number of possible unintended adverse effects posed by IoTs for OSH purposes, one should seriously ask the question whether the deployment of these devices should be encouraged or refrained. For such an assessment, the principle of proportionality is very important theoretical tool. It may not be easy to apply it in the case of IoTs because the positive and negative or unintended impacts to be compared in the proportionality evaluation are often of a different nature (economic costs and benefits, physical or psychological effects, levels of trust, etc.) and are also unevenly distributed between the different types of stakeholders (employers, workers, third parties). Therefore, one should be aware that large margins of interpretation remain possible. A second difficulty is the fact that numerous and interconnected effects are at stake and are often difficult to disentangle. Overall, however, we feel that a fair application of the proportionality principle makes it difficult to take a positive view of an IoT deployment for OHS purposes. At minima, it highlights the urgent need for nuanced assessments before deploying such technologies in complex situations.

Our results need also to be read in comparison with the results of a connected empirical study that we have conducted (36). In that study, we used focus groups and individual interview to obtain first hand stakeholder points of view. Our results reveal further ethical highlights that were not explicitly or only marginally discussed in the academic literature. Notably, numerous stakeholders that we interviewed doubted that (in most cases) IoTs could efficiently replace classical OSH tools and procedures. This worry comes ahead of discussing proportionality issues. A linked issue is the difficulty of setting up efficient safeguards (to avoid risks of excessive surveillance, data security, etc.) without compromising the adequacy of the devices. Interviewees were also attentive to issues specifically attached to the roles of people within a company. They worried, for instance, about the competencies and integrity of human resources services while handling data produced by IoTs for OSH purposes. They elaborated on the conflicts of interest of the staff involved: on the one hand, occupational physicians or OSH specialists need to support employees and IoT may help them in this task, on the other hand, they obtain data that are valuable (e.g., for managerial purposes) to their employer. Interviewees also discussed the uncomfortable situation in which direct managers can be placed when they must deploy ethically questionable IoTs within their working unit. Further, they wondered about employers’ (existing of new) obligations towards workers when IoTs for OSH purposes are deployed.

Furthermore, our data show the field’s dynamic and emphasises the need to situate expressed ethical concerns in a time and political perspective (40, 44). Academic discussions on the implications of 4.0 and 5.0 technology have lately become abundant, as we can see by the articles’ publication dates. Even though we used 2008 as the starting date for the review, nearly 90% of the articles we found were published in the last 5 years. One observable trend in this growing literature is scholars’ interest in the topic of data, privacy and access to own individual information. This selective ethical attention is probably a result of the adoption of the European Data protection regulation (37). In contrast, other topics are overlooked or only mentioned briefly, although they would deserve more development. This is the case with the emerging environmental impact discussion, which is likely to become a major theme in the coming years in most domains of technology (1, 35). Another important topic, overlooked in the IoT literature but highlighted in discussions about AI in medicine, is the suboptimal continuous assessment and maintenance plans of the technology: outdated devices pose all sorts of risks to users (74, 75).

The dissonance between our research results and the ongoing development of ethical awareness also highlights the limits of the classical preventive approach based on risk analysis, which dominates the regulation of information technology. Scholars have already highlighted the limits of such an approach and the difficulty of anticipating what is unknown (25). Our study shows that short and sometimes automatized checklists of risks are insufficient regulatory solutions for the deployment of AI technology, and in particular for assessing IoTs for OSH purposes. Stakeholders need to stay in charge and should be able to use critical thinking before making decisions about the deployment of such technology.

## 6 Conclusion

This paper shows that deploying of IoTs for OSH generates an unexpected and substantial list of ethical issues. Moreover, the analysis reveals two under-investigated categories of ethical issues, which cover the impact of adverse effects and contextual factors. In this regard, the paper enlightens decision-makers, occupational health professionals, and end-users (employees) who are unlikely to be aware of all relevant issues. Overall lack of ethical awareness is confirmed by our complementary qualitative study (36), in which we found that relevant stakeholders fail to spot numerous and important issues discussed in the academic literature. Such blind spots are even noticeable in the academic realm since several articles that we reviewed heavily focused on the positive aspects of the technology. None of the more critical articles that we reviewed spotted all the issues listed here. Therefore, this paper offers an analytical grid of five categories that could serve as a source of information for the selection and improvement of existing ethical frameworks [e.g., (10, 17, 23, 40, 63, 74, 76–78)], as teaching material for the training of OSH professionals and occupational physicians, as a springboard for targeted scientific research on the use of IoTs in the area of occupational medicine, and as a call to improving laws and regulations.

This paper paves the way for further research. First, it calls for conducting in-depth studies on the ethical issues briefly listed in the result section. These analyses could be connected to related discussions about AI in medicine. Overall, we think that more critical research should be conducted on the way of elaborating and conducting ethical assessments of evolving technologies. Regulators develop guidelines and frameworks for fostering the deployment of AI systems, but they should give greater consideration to the societal and contextual impact of technology, even of technology originally deployed for good purposes. To conclude, this study’s main message is that much more can go wrong than an intelligent and careful stakeholder or academic expert might expect at first glance.

## Data Availability

The original contributions presented in the study are included in the article and in the Supplementary File 1, further inquiries can be directed to the corresponding author.
