# Supplementary material for: Behind the good of digital tools for occupational safety and health: a scoping review of ethical issues surrounding the use of the internet of things
Source: Front Public Health. 2024 Dec 20;12:1468646. doi: 10.3389/fpubh.2024.1468646 (PMC11719680; doi:10.3389/fpubh.2024.1468646)
Supplement: Supplementary file 1 [file Supplementary_file_1.docx]

Behind the Good of Digital Tools for Occupational Safety and Health: a Scoping Review of Ethical Issues Surrounding the Use of the Internet of Things

## Supplementary file

| **Supplementary Table 1.** Selection of the most meaningful search queries of the scoping review. The “items kept” were selected with the following eligibility criteria: [published in a peer-reviewed journal] *AND* [discusses ethical opportunities and issues of IoT] *AND* [includes an explicit discussion on OSH] *OR* [discusses with some detail at least one case of IoT used for OSH purposes]. | | | |
| --- | --- | --- | --- |
| **Database** | **Search query** | **Items returned** | **Items kept** |
|  | + filter “published up to 2008” |  |  |
| Webofscience | technology *AND* ethics *AND* workplace | 231 | 8 |
|  | ethic *AND* occupational health *AND* technology | 62 | 6 |
|  | algorithms *AND* health *AND* work ethic* | 498 | 3 |
|  | algorithms *AND* health *AND* workplace | 290 | 3 |
|  | artificial *AND* intelligence *AND* ethic *AND* workplace | 26 | 3 |
|  | AI *AND* ethics *AND* workplace | 26 | 2 |
| Philpapers | artificial intelligence & ethic & workplace | 11 | 1 |
|  | ethic & work & health & technology | 133 | 2 |
|  | ethic & internet of things \| IoT \| AI \| Technology \| big data \| health \| work \|algorithms  *(“*&*” is an equivalent to “*AND*” and “*\|*” to “*OR*”)* | 126 | 1 |
| Googlescholar | “internet of things” *AND* “ethics” *AND* “workplace” *AND* “occupational health” | 200 first outputs (among 1020) | 15 |
|  | “internet of things” *AND* “ethics” *AND* “workplace” *AND* “occupational health” + filter “review articles” | 99 | 11 |
|  | (ethic* *OR* moral*) *AND* (“internet of things” *OR* “IoT” *OR* "wearable*”) *AND* (“occupational health” *OR* “health monitoring” *OR* “safety”) + filter “review articles” —> search query inspired by Mittelstadt 2017 | 200 first outputs (among 12200) | 21 |
| **Total excluding duplicates** (i.e. excluding items that came up in several research queries) | | **1495** | **47** |
